# Relationships Between Base-Catalyzed Hydrolysis Rates or Glutathione Reactivity for Acrylates and Methacrylates and Their NMR Spectra or Heat of Formation

**DOI:** 10.3390/ijms13055789

**Published:** 2012-03-13

**Authors:** Seiichiro Fujisawa, Yoshinori Kadoma

**Affiliations:** 1 Meikai University School of Dentistry, Sakado, Saitama 350-0283, Japan; E-Mail: fujisawa33@nifty.com; 2 Institute of Biomaterials and Bioengineering, Tokyo Medical and Dental University, Kanda-surugadai, Chiyoda-ku, Tokyo 101-0062, Japan

**Keywords:** acrylate and methacrylate esters, base-catalyzed hydrolysis, GSH reaction rate constants, ^13^C NMR spectra, heats of formation, QSPRs

## Abstract

The NMR chemical shift, *i.e*., the π-electron density of the double bond, of acrylates and methacrylates is related to the reactivity of their monomers. We investigated quantitative structure-property relationships (QSPRs) between the base-catalyzed hydrolysis rate constants (*k*1) or the rate constant with glutathione (GSH) (log *k*_GSH_) for acrylates and methacrylates and the ^13^C NMR chemical shifts of their α,β-unsaturated carbonyl groups (δC_α_ and δC_β_) or heat of formation (Hf) calculated by the semi-empirical MO method. Reported data for the independent variables were employed. A significant linear relationship between *k*1 and δC_β_, but not δC_α_, was obtained for methacrylates (*r*^2^ = 0.93), but not for acrylates. Also, a significant relationship between *k*1 and Hf was obtained for both acrylates and methacrylates (*r*^2^ = 0.89). By contrast, log *k*_GSH_ for acrylates and methacrylates was linearly related to their δC_β_ (*r*^2^ = 0.99), but not to Hf. These findings indicate that the ^13^C NMR chemical shifts and calculated Hf values for acrylates and methacrylates could be valuable for estimating the hydrolysis rate constants and GSH reactivity of these compounds. Also, these data for monomers may be an important tool for examining mechanisms of reactivity.

## 1. Introduction

**Figure 1:**
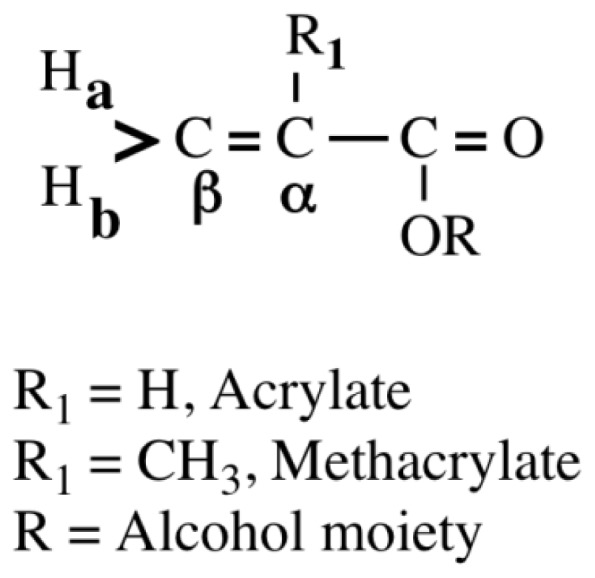
The structure of acrylates and methacrylates.

Acrylates and methacrylates (Figure 1) are widely used in the formation of polymeric materials for medical, dental and industrial applications.

Many studies have investigated the hydrolysis reaction of acrylates and methacrylates [1–6] because the hydrolysis of monomers and decomposition of cured polymers are implicated in environmental pollution and toxicity. The hydrolysis reaction is one of the major sources of degradants (or metabolites) in *in vitro* and *in vivo* biodegradation tests stipulated in the Chemical Substance Control Law [7]. Therefore, reliable and economical methods are needed for predicting the hydrolysis rates of the large number of these chemicals. Freidig *et al*. have previously reported that when acrylates and methacrylates with α,β-unsaturated carbonyl groups undergo hydrolysis in alkaline media, their electrophile (carbon: α-carbon, β-carbon, carbonyl carbon (>C=O) may be preferentially attacked by a nucleophile (water, hydroxyl anion (OH^−^), glutathione (GSH) thiyl radical (GS^−^) [6]. Double bonds (C=C) in acrylates and methacrylates act as an active center for Michael addition and radical scavenging oxidation because HOMO (Highest Occupied Molecular Orbital) and LUMO (Lowest Unoccupied Molecular Orbital) in methacrylate molecules exist in the double bonds [8]. Also, since it is well known that the alkaline-mediated hydrolysis of acrylate polymers is due to an attack of OH^−^ on the intermolecular carbonyl carbon in the ester group, the hydrolysis rate constant of a monomer is probably linked to that of the corresponding polymer and their reactions proceed via a similar mechanism. Therefore, we hypothesized that the OH^−^-catalyzed hydrolysis of acrylates and methacrylates may have an influence on the NMR-chemical shift of their β-carbon (δC_β_), because OH^−^ could attack the β-carbon in a monomer molecule in the initial stage through a hydrolysis reaction. Subsequently, through a reaction involving the B_AC_2 mechanism (*i.e*., through nucleophilic attack on the >C=O group) in most cases, acrylates and methacrylates are decomposed into acrylic acid and methacrylic acid together with alcohols, respectively, through hydrolysis. The resonance stabilization of methacrylates is dependent on their reactivity with these compounds. Kuznetsova *et al*. [9] previously investigated the hydrolysis of *N*,*N*-dimethylaminoethyl methacrylate via the A_AC_2 and B_AC_2 mechanism, and found that the monomer was hydrolyzed to methacrylic acid and amino alcohol in an anion, pH, and surfactant-dependent manner [9]. Acrylate and methacrylate esters are more highly hydrolyzed with a base than with an acid [1,6,9], but their hydrolysis rate constants have been reported to be considerably small under alkaline conditions at 20 °C or 30 °C [1–4,6]. There are a few QSPRs for acid- or base-catalyzed ester hydrolysis of methacrylates [2,4,6], however the limited number of monomers have been chosen for QSARs study [1–4,6] because the hydrolysis rate for many hydrophobic homologous acrylate and methacrylate esters may be below the detection limit of assays. We previously reported that the glutathione (GSH) reactivity of acrylates and methacrylates was significantly related to their δC_β_, possibly due to the high reactivity of GS^−^ with C_β_ of these monomers [10–12].

Mallik and Das [2] previously investigated the kinetics of alkaline hydrolysis for methyl and ethyl acrylates and their corresponding methacrylates, and revealed a possible relationship between the hydrolysis rate constants and the experimentally determined activation energy for these compounds. Also, Nakajima *et al*. [13] investigated the relationship between the acid-catalyzed hydrolysis rates for acetates and acrylates and their activation energy calculated using the MOPAC program, and found a good relationship between the hydrolysis rate constant and calculated activation energy for these compounds, except for formates and methacrylates, which showed a large discrepancy between the experimental and calculated values. The authors mentioned that recalculation for formates and methacrylates was desirable whenever possible. If a more precise calculation for monomers is required, this could be one way of utilizing a higher level of theory, such as density function theory or *ab initio* MO calculation.

In order to clarify the base-catalyzed hydrolysis reaction for acrylates and methacrylates in the light of currently available data, we investigated the relationship between the base-catalyzed hydrolysis rate constants or reaction rate constants with the GSH and ^13^C NMR chemical shifts (δC_β_) or heat of formation (Hf) for these compounds. Previously reported data [14,15] for the independent variables were used.

## 2. Results and Discussion

### 2.1. Hydrolysis

#### 2.1.1. δC_β_ Parameter

Previously reported data for base-catalyzed hydrolysis constants (*k*1, *k*3) are shown in Tables 1 and 2. ^13^C NMR chemical shifts for the α,β-unsaturated carbonyl group of acrylates and methacrylates are shown in Table 1. The ^13^C NMR chemical shift of the β-carbon (δC_β_) of monomers is also quantitatively related to the π-electron density. The higher the π-electron density on the β-carbon, the higher the magnetic field where the NMR peak is observed; that is, as the π-electron density increases, the chemical shift value (δ) becomes smaller [14]. Therefore, if OH^−^ attacks the C_β_ in the monomer molecule, it should be possible to correlate the magnitude of the shift with the reactivity of the monomers.

We investigated the relationship between *k*1 and δC_β_ or δC_α_ for both acrylates and methacrylates. There was no relationship between *k*1 and δC_β_ or δC_α_ for both acrylates and methacrylates. By contrast, a significant relationship between *k*1 and δC_β_, but not δC_α_, was obtained when acrylates and methacrylates were separated, particularly in the latter situation. Equation (1) for methacrylates is given as follows:

(1) $$k1=-17.118\;(\pm 0.130)+0.137\;(\pm 0.021)\;\delta {\text{C}}_{\beta }\;(n=5,r^{2}=0933,p<0.01)$$

The *k*1 value for methacrylates was calculated using this equation. The results are also shown in Table 1. The calculated *k*1 values for isoPMA and isoBMA with the branched substituent were less than the corresponding experimental values. By contrast, the calculated *k*1 values for MMA and allyl MA were larger than the experimental values. Also, the *k*1 value for nBMA could not be determined using Equation (1), and the value of δC_β_ was below the limit of detection using that equation. A base-catalyzed second-order hydrolysis rate constant of 2.7 × 10^−3^ L/mole-s was estimated using a structure estimation method [16]. The hydrolysis rate constant for nBMA appeared to be less than that for isoBMA, as the δC_β_ for nBMA was smaller than that for isoBMA. We predicted the *k*1 value using Equation (1) for some monomers that have been used as medical, dental and industrial materials. The *k*1 values (mol^−1^s^−1^) for ethyleneglycol dimethacrylate (EGDMA), triethyleneglycol dimethacrylate (TEGDMA) and MMA were 0.130, 0.062 and 0.034, respectively. Note that the δC_β_ values (ppm) for EGDMA, TEGDMA and MMA were 125.9, 125.5, and 125.2, respectively [11]. Dimethacrylates such as EGDMA and TEGDMA appeared to be more hydrolyzed than MMA, a monomethacrylate. This may be due to the fact that dimethacrylates possess two double bonds in the molecule that has an affinity to OH^−^. We previously investigated the changes in the ^13^C NMR chemical shift for methacrylates induced by their interaction with the phospholipid liposome system in deuterium oxide (D_2_O) at 30 °C, and it was found that the changes in chemical shifts of electrophiles (carbons: β-carbon, and carbonyl carbon (>C=O)) for TEGDMA and EGDMA, particularly for the latter, was 3–5 fold larger than those for the corresponding MMA; the chemical shifts of β- and carbonyl carbon in the dimethacrylate molecule shifted to a higher field in liposomes in D_2_O, compared to those in MMA molecule [18]. Although these finding may not be directly related to hydrolysis of methacrylates, it has been assumed from the shielding of chemical shift of β- and carbonyl carbon in D_2_O that the hydrolysis rate of dimethacrylates is greater than that of MMA. The higher the reactivity, the higher the field in which β-carbon resonates.

On the other hand, there was no relationship between *k*1 or *k*3 and δC_β_ for both acrylates and methacrylates. In general, the electron-releasing methyl group was expected to reduce the rate of alkaline hydrolysis. Indeed, the *k*1 value for isoBA was greater than that for isoBMA. Similarly, the *k*1 value for EA was greater than for the corresponding EMA (Table 1).

**Figure 2:**
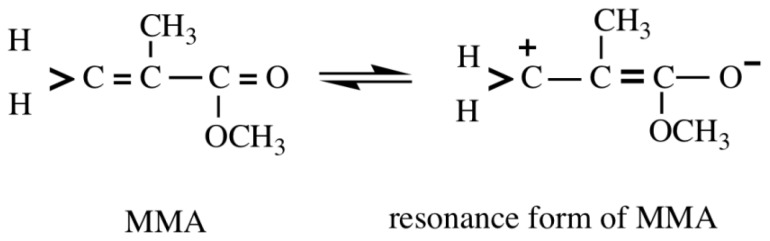
The resonance form of MMA [14]. The double bonds of MMA monomers change to single bonds, and the diamagnetic anisotropy effect of the carbonyl group at the vinylidene proton is enhanced. As the resonance effect becomes important, the double bond character of CH_2_=C decreases.

The chemical shifts of the proton attached to the β-carbon of monomers are linearly related to the corresponding value of δC_β_, where Ha represents the proton *trans* to the substituent and Hb the proton *cis* to that. The δHa for acrylates and methacrylates was related to their δC_β_ much more than the δHb. An understanding of the chemical shift difference between Ha and Hb, |δHa – δHb|, may be necessary to clarify the difference in the hydrolysis rate constants for acrylates and methacrylates. Table 2 shows Ha, Hb and |δHa – δHb|. We investigated the relationship between |δHa – δHb| and δC_β_ for the separation of the acrylates and methacrylates. There was a significant relationship between |δHa – δHb| and δC_β_ for acrylates (*n* = 7, *r*^2^ = 0.850, *p* < 0.01), whereas there was no such relationship for methacrylates when the training set shown in Table 2 was used. Because of the electron-withdrawing character of the carbonyl group in monomers, resonance stabilization increases the electron density at the carbonyl carbon (>C=O). As an example, MMA and its resonance form are shown in Figure 2. The π-electron density of the α,β-unsaturated carbonyl groups could be responsible for the resonance stabilization.

As shown in Table 2, the charge density and |δHa – δHb| value for acrylates were greater than those for methacrylates, suggesting a possible relationship between the electron density at the carbonyl carbon and the |δHa – δHb| value. These findings can be interpreted in terms of a relationship between the base-catalyzed hydrolysis reaction and the δC_β_ value for methacrylates and acrylates. For the chemical shifts (Ha, Hb) of the proton attached to β-carbon, the coefficient is much closer to unity for the chemical shift of Ha than that of Hb, probably due to the fact that the chemical shift of Hb is more strongly affected by the diamagnetic anisotropy of the substituent. From the geometrical consideration on the molecular model, differences in the deshielding effects of the C=O group are larger at Hb than at Ha [14]. As the resonance stabilization becomes more important, the |δHa – δHb| becomes larger; the |δHa – δHb| is related to *Q* which is a measure of resonance stabilization. *Q* is a parameter concerning a monomer based on the *Q–e* scheme. As compared with the hydrolysis rate constant (*k*1) between the acrylate esters and the corresponding methacrylate esters (Table 1), the rate constant for EA is greater than the EMA one, and similarly, that for isoBA is greater than the isoBMA one.

Next, we investigated the relationships between the base-catalyzed hydrolysis rate constant (*k*3) and δC_α_ or δC_β_ for the test compounds, acrylates (methyl acrylate, ethyl acrylate, butyl acrylate) and methacrylate (MMA) (Table 1). With the exception of MMA, significant linear relationships of *k*3 with δC_α_ and δC_β_ were found, the correlation being *r*^2^ = 0.97 and *r*^2^ = 0.98, respectively. An increase of *k*3 occurred when δC_β_ increased, whereas *k*3 increased as δC_α_ decreased. Although the hydrolysis rates were determined for only a limited number of acrylates (*n* = 3), this finding indicated that *k*3 increased along with the π-electron density for C_α_. Thus in acrylates, the preferential site of attack of OH^−^ may be both the C_α_ and C_β_, which is possibly due to the absence of α-methyl substituent in the acrylate molecule. However, further studies will be needed to clarify the details of ester hydrolysis of acrylates in alkaline media.

The hydrolysis rates of chemicals are reportedly correlated with Hammet σ, the Taft parameters, σ* and Taft steric property parameter, E(s), and the van der Waals radius [6,19]. Freidig *et al*. reported previously that the base-catalyzed hydrolysis of six methacrylates was able to establish a linear free energy relationship as a function of the Taft σ* but not of the Taft E(s) [6]. Therefore, we examined the relationships for the δC_β_ *vs*. Taft σ* for the six monomers, and it was found that a good relationship between the two descriptors was obtained (*r*^2^ = 0.9, *p* < 0.01) (data not shown). This clearly indicated that the attack of OH^−^ on the β-carbon of methacrylates may be also associated with the electronic effects of the substituents (R, alcohol moiety as shown in Figure 1). The chemical shifts δC_β_ for methacrylates likely reflect their electronic effects of the substituents. The π density shown in the NMR spectra is thus considered to be a useful parameter.

#### 2.1.2. Hf Parameter

The *k*1, *k*2, Hf and ΔH_f_° values for acrylates and methacrylates are shown in Table 3.

Next we investigated the relationship between *k*1 and Hf, and found that it was significant (*r*^2^ = 0.89, *p* < 0.05). Equation (2) is given as follows:

(2) $$k1=0.2288\;(\pm 0.015)+0.0026\;(\pm 0.001\;)\;\text{Hf}\;(n=5,r^{2}=0.891,p<0.05)$$

Data on the physical and thermodynamic properties of vinyl monomers are needed for the design and operation of industrial chemical processes. Vatani *et al.* previously predicted the standard enthalpy of formation using a QSPR model [21]. The application of this model may be useful for evaluating the biological activity of various vinyl monomers used for medical, dental and industrial materials. We investigated the relationship between the calculated Hf and the ΔH_f_° reported in DIPPR 9801 [20] for acrylates and methacrylates using the data set shown in Table 3, and found a good linear relationship between the two independent variables (*n* = 10, *r*^2^ = 0.992, *p* < 0.001).

The relationship between *k*1 and ΔH_f_° for both acrylates and methacrylates is given as Equation (3) as follows:

(3) $$k1=0.211(\pm 0.006)+0.00044(8.1\times 10^{5})\;\Delta {\text{H}}_{\text{f}}{}^{\circ}\;(n=5,r^{2}=0.907,p<0.05)$$

The *k*1 value in the ΔH_f_° term for acrylates and methacrylates was calculated from Equation (3), and the results are shown in Table 3. The experimentally determined and calculated values of *k*1 for each monomer were similar. We then investigated the relationship between the rate constant (*k*2) and Hf for MA, EA, MMA and EMA. Equation (4) is given as follows:

(4) $$k2=0.085\;(\pm 0.016)+0.0013\;(\pm 0.000)\;\text{Hf}\;(n=4,r^{2}=0.941,p<0.05)$$

The calculated *k*2 value is also shown in Table 3. These findings indicated that the *k*1 or *k*2 value for both acrylate and methacrylate esters was related to their Hf. In the present study, the hydrolysis rate constant for acrylate esters with the simple alkyl substituent was higher than for the corresponding methacrylate esters. It was concluded that the Hf (or ΔH_f_°) value for acrylates and methacrylates is valuable for the estimation of the rate constants. As the Hf (or ΔH_f_°) value for monomers increased, their hydrolysis rate constant increased. The hydrolysis rate-determining process for acrylates and methacrylates may be controlled by their Hf (or ΔH_f_°) value.

### 2.2. GSH Reactivity

Acrylate and methacrylate esters are important chemicals in the polymer industry, and their induction of toxicity is considered to involve alkylation of crucial cellular nucleophiles through the Michael reaction [22]. We investigated the relationship between *k*_GSH_ and δC_α_ or δC_β_ for both acrylates and methacrylates, and obtained a good correlation for δC_β_. For the δC_β_, Equation (5) is given as follows:

(5) $$\text{log}\;k_{\text{GSH}}=-56.155\;(\pm 0.131)+0.443(\pm 0.019)\delta {\text{C}}_{\beta }\;(n=8\;r^{2}=0.989,p<0.001)$$

By contrast, there was a weak correlation for δC_α_ (*r*^2^ = 0.65). It was assumed from this that the preferential site of GS^−^ attack for these monomers would likely be the β-carbon. We calculated log *k*_GSH_ using Equation (5) and the result is also shown in Table 1. As the δC_β_ for monomers increased, their log *k*_GSH_ increased. This showed that π-electron density of β-carbon for both acrylates and methacrylates may play an important role in the rate-determining process in GSH reactivity. GSH reactivity for acrylates was considerably higher than for methacrylates. It is interesting to note that the log *k*_GSH_ for both acrylates and methacrylates was not related to their Hf. Freidig *et al.* reported that hydrolysis of acrylates does not interfere with the GSH reactivity assay [6]. In the present study, the base-catalyzed hydrolysis rate constant for acrylates and methacrylates was correlated with their Hf value (Equations (3) and (4)), whereas conversely, their GSH reactivity was not correlated with the Hf value. These findings support those of the above study [6].

Putz *et al.* [23] determined the actual quantitative-structure activity relationships (QSARs) for biological activity using the parameters recommended by the Hansch group [24] (hydrophobicity, polarizability and total energy) and special reactivity indices (electronegativity (χ) and chemical hardness (η)) employing computational chemistry. The need for these parameters has potentially been met by QSARs and quantitative structure-property relationships (QSPRs), and the total enthalpy of formation as an independent variable is useful for kinetic studies of the hydrolysis reaction of vinyl monomers. We recently reported a good QSAR for biological activities *vs*. theoretical parameters for vinyl monomers [12] and phenolic compounds [25]. On the other hand, to develop the phenolic carbonate ester prodrugs, Østergaard and Larsen investigated the water, acid and base catalyzed hydrolysis rate constants for various carbonate esters with fatty acid-like structures, bioreversible derivatives of phenols [26]. They reported that the hydrolysis rate constant of such chemicals may be useful for estimating their physical properties favorable for drug transport to the target site within the body.

## 3. Experimental Section

### 3.1. Monomers

The monomers used are abbreviated as follows. Acrylates: methyl acrylate (MA), ethyl acrylate (EA), *n*-butyl acrylate (nBA), isobutyl acrylate (isoBA), hexyl acrylate (Hexyl A); Methacrylates: methyl methacrylate (MMA), ethyl methacrylate (EMA), isopropyl methacrylate (isoPMA), isobutyl methacrylate (isoBMA), allyl methacrylate (allyl MA), benzyl methacrylate (benzyl MA).

### 3.2. NMR Spectra

The ^13^C NMR chemical shift data for various monomers in chloroform-*d* (CDCl_3_) were taken from the literature [14]. Briefly, the chemical shifts of the indicated monomers were measured in CDCl_3_ at 35 °C at 125 and/or 500 MHz, respectively, using tetramethylsilane (TMS) as an internal standard.

### 3.3. Hydrolysis

The hydrolysis rate constants (*k*1) for acrylate EA, HA, isoBA and methacrylates (MMA, allyl MA, benzyl MA, isoPMA, isoBMA) under alkaline conditions (pH 10) were taken from Freidig *et al*. [6]. The hydrolysis rate measurements were carried out using an HPLC method at 20 °C. Also, the alkaline hydrolysis rate constant *k*2 (second-order rate constant) for MA, EA, MMA and EMA at 30 °C was taken from Mallik and Das [2]. Furthermore, the second-order rate constant (k3) data for base-catalyzed acrylates (MA, EA, BA) and methacrylates (MMA) obtained using a diffusion method were taken from Sharma and Sharma [4].

### 3.4. Heats of Formation (Hf) and Standard Enthalpy of Formation (ΔH_f_°)

For calculation of the molecular HF descriptor, the optimized chemical structures of compounds are needed. We have previously calculated the Hf values for acrylates and methacrylates, and those data were used in the present work [10,11,15]. Briefly, calculations of Hf were performed using the PM3/CONFLEX method. To obtain fine geometry details in the present study, initial geometry optimization was performed using CONFLEX5 (Conflex, Tokyo, Japan), then calculations using the PM3 method in the MOPAC 2000 program were carried out on a Tektronix CAChe workstation (Fujitsu Ltd., Tokyo, Japan). Also, the ΔH_f_° values for acrylates and methacrylates were taken from the literature [20,21].

### 3.5. GSH Reactivity

Data for GSH reactivity were taken from the literature [6]. The reaction rate constant (*k*_GSH_) was measured at 20 °C at pH 8.8.

### 3.6. Multi-Regression Analysis

The multi-regression equations were calculated using StatMate III (ATMS Co., Ltd., Tokyo, Japan).

## 4. Conclusions

The present study has shown that the base-catalyzed hydrolysis rate constant for methacrylates is significantly related to δC_β_. By contrast, there was no relationship between two independent variables for acrylates. A good relationship for the hydrolysis rate constant *vs*. heat of formation was also obtained for both acrylates and methacrylates. The GSH reactivity for both acrylates and methacrylates was related to δC_β_, but not to Hf. The NMR spectra and heat of formation for acrylates and methacrylates could be used to estimate the base-catalyzed hydrolysis rate constants and GSH reactivity of these compounds, and also may be an important tool for examining the mechanism of their reactivity.

## Figures and Tables

**Table 1:** Base-catalyzed hydrolysis rate constants (*k*1, *k*3), reaction rate constants with glutathione (GSH) for acrylates and methacrylates and the ^13^C NMR chemical shifts of their β-carbon (δC_β_) and α-carbon (δC_α_).

Monomer^a^	^13^C NMR Chemical Shifts		Reported *k*1 at 20 °C (mol^−1^·s^−1^)^c^	Calculated from Equation (1)	Reported *k*3 at 30 °C (mol^−1^·s^−1^)^d^	Reported log *k*_GSH_ (mol^−1^·min^−1^)^c^	Calculated from Equation (5)

	ppm^b^ δC_β_	ppm^b^ δC_α_					
MA	130.56	128.15	–	–	0.198	–	1.68
EA	130.24	128.59	0.050	–	0.102	1.6	1.54
nPA	130.22	128.57	–	–	–	–	1.53
nBA	130.21	128.61	–	–	0.074	–	1.53
isoBA	130.23	128.6	0.020	–	–	1.6	1.54
HA	130.23	128.63	0.087	–	–	1.3	1.54
MMA	125.23	136.15	0.026	0.039	0.083	−0.7	−0.68
EMA	124.97	136.51	–	0.003	–	–	−0.79
isoPMA	124.95	136.52	0.008	0.0002	–	−1.0	−0.8
nBMA	124.7	136.41	(0.0027)^e^	nd	–	–	−0.91
isoBMA	124.98	136.52	0.007	0.0043	–	−0.73	−0.79
Benzyl MA	125.66	136.21	0.110	0.097	–	−0.49	−0.49
Allyl MA	125.46	136.23	0.059	0.070	–	−0.52	−0.58

a For abbreviations see the text;

b Taken from Reference [14];

c Taken from Reference [6];

d Taken from Reference [4];

e Taken from Reference [16];

nd: not determined.

**Table 2:** ^1^H NMR chemical shifts for acrylates and methacrylates and the charge density of their carbonyl carbon.

	^1^H NMR Chemical Shifts^b^					Charge Density^e^ (C=O) a.u.
		
Monomer^a^	Ha ppm	Hb ppm	H ppm	|δHa–δHb|^c^ ppm	Q^σ^(C=O)^d^	
MA	5.825	6.406	0.581	0.581	0.1666	–
EA	5.807	6.395	6.113	0.587	0.1662	1.06
nPA	5.809	6.397	6.127	0.588	–	–
nBA	5.805	6.391	6.119	0.586	0.1662	
isoBA	5.813	6.4	6.113	0.587	0.1662	1.04
Hexyl A	5.804	6.391	6.12	0.587	–	1.03
Benzyl A	5.83	6.349	6.162	0.519	–	–

MMA	5.55	6.1	–	0.55	0.1638	0.94
EMA	5.541	6.09	–	0.555	0.1634	–
nPMA	5.54	6.1	–	0.56	–	–
nBMA	5.532	6.091	–	0.559	0.1634	–
isoBMA	5.543	6.108	–	0.565	–	0.91
Benzyl MA	5.572	6.153	–	0.581	–	0.88
Allyl MA	5.574	6.138	–	0.564	–	0.94
Hexyl MA	5.537	6.092	–	0.555	–	–

a For abbreviations see the text;

b Taken from Reference [14];

c The chemical shift difference between Ha and Hb;

d Taken from Reference [17];

e Taken from Reference [6].

**Table 3:** Reported data for base-catalyzed hydrolysis rate constants (*k*1, *k*2) and heat of formation (Hf, ΔH_f_°) for acrylates and methacrylates and their rate constants calculated using the relevant Equations.

Monomer^a^	Heat of Formation (Hf) (kcal mol^−1^)^b^	Enthalpy of Formation (ΔH_f_°) (kJ mol^−1^)^c^	Reported *k*1 (mol^−1^sec^−1^)^d^ 20 °C	Calculated from Equation (2)	Calculated from Equation (3)	Reported *k*2 (mol^−1^s^−1^)^e^ 30 °C	Calculated from Equation (4)
MA	−67.387	−362.2	–	0.054	0.052	0.015	0.016
EA	−72.173	−379.59	0.05	0.040	0.044	0.013	0.011
nPA	−77.404	−407.17	–	0.025	0.029	–	0.005
nBA	−82.791	−433.45	–	0.011	0.020	–	nd
isoBA	−82.435	−438.95	0.02	0.012	0.018	–	nd
MMA	−74.768	−399.13	0.026	0.032	0.036	0.008	0.009
EMA	−79.542	−421.34	–	0.020	0.026	0.003	0.003
nPMA	−84.767	−446.7	–	0.006	0.014	–	nd
nBMA	−90.156	−471.39	–	nd	0.0004	–	nd
isoBMA	−89.832	−465.16	0.007	nd	0.006	–	nd
Benzyl MA	−49.295	–	0.11	0.1	0.091	–	0.021

a For abbreviations see the text;

b Taken from Reference [11];

c Taken from Reference [20];

d Taken from Reference [6];

e Taken from Reference [2].
